# Supplementary material for: The microbiome biomarkers of pregnant women’s vaginal area predict preterm prelabor rupture in Western China
Source: Front Cell Infect Microbiol. 2024 Oct 31;14:1471027. doi: 10.3389/fcimb.2024.1471027 (PMC11560878; doi:10.3389/fcimb.2024.1471027)
Supplement: Supplementary file 1 [file DataSheet1.zip › compare_1/Community/Pieplot/phylum/sample/P21.phylum.pieplot.pdf]

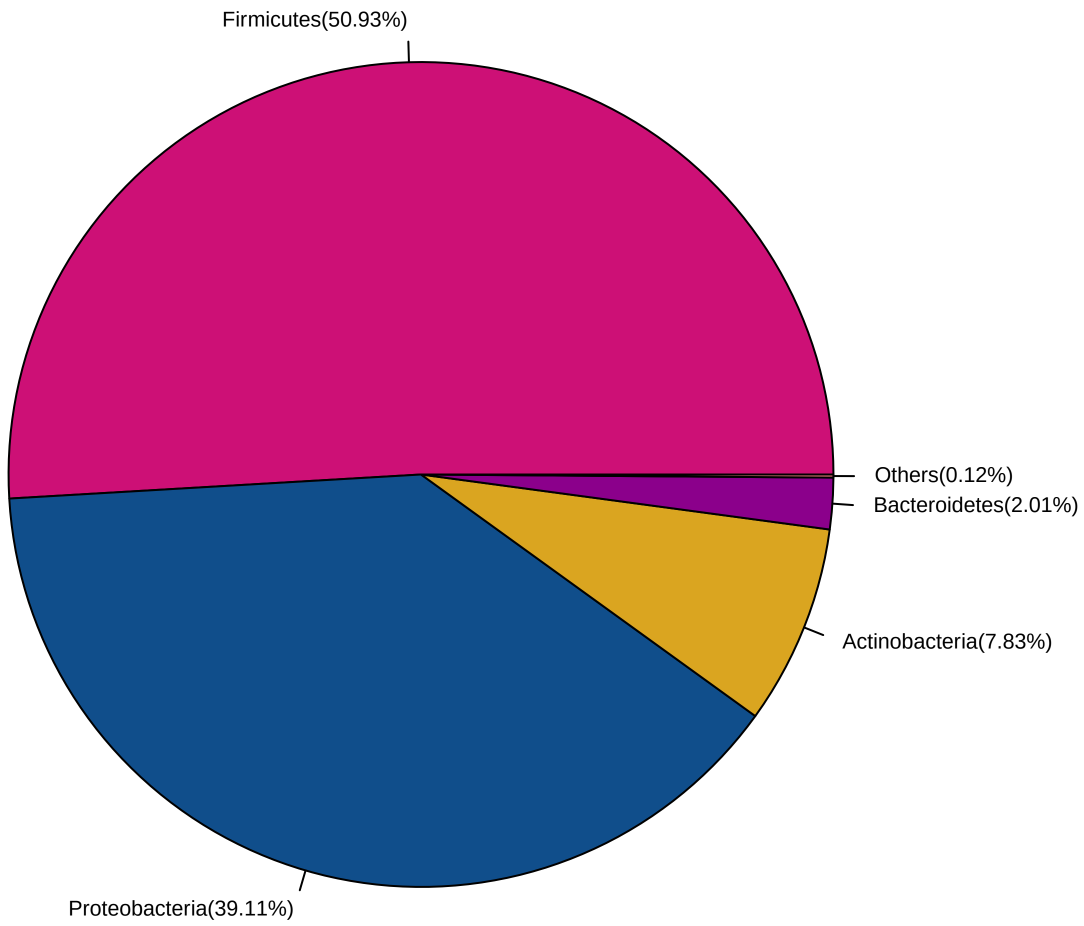
Firmicutes(50.93%)
Others(0.12%)
Bacteroidetes(2.01%)
Actinobacteria(7.83%)
Proteobacteria(39.11%)
